# Walking Environment and Obesity: A Gender-Specific Association Study in Shanghai

**DOI:** 10.3390/ijerph19042056

**Published:** 2022-02-12

**Authors:** Hei Gao, Zike Xu, Yu Chen, Yutian Lu, Jian Lin

**Affiliations:** 1 The Architectural Design & Research Institute of Zhejiang University Co., Ltd., Zhejiang University, Hangzhou 310028, China; gh1@zuadr.com (H.G.); cyzuadr@126.com (Y.C.); 2 Center for Balanced Architecture, Zijingang Campus, Zhejiang University, Hangzhou 310058, China; 3 College of Agriculture and Biotechnology, Zhejiang University, Hangzhou 310058, China; 22016271@zju.edu.cn; 4 College of Computer Science and Technology, Zhejiang University, Hangzhou 310058, China; yutianlu@zju.edu.cn; 5 Department of Urban and Regional Planning, College of Urban and Environmental Sciences, Peking University, Beijing 100871, China

**Keywords:** obesity, BMI index, walking environment, gender

## Abstract

Walking environment is commonly cited as an element that reduces the risk of obesity. Many literatures have shown that the impact of walking environment on the incidence rate of obesity may vary across gender, but few studies have conducted in-depth investigations. The present study aimed to provide empirical evidence for a cross-sectional association between the built community environment and the incidence of obesity among male and female residents. Thus, we collected height and weight level of 1355 residents and constructed seven walking environment indicators around 54 communities. Also, BMI was calculated and categorized to define overweight and obesity. We used generalized estimation equation to evaluate the gender-specific association between walking environment on obesity based on a diverse population sample. The study showed that female residents who lived in neighborhoods with higher road sky view index (*p* = 0.033; OR = 0.002 [95% CI = 0.001–0.619]) and increased intersection density (*p* = 0.009; OR = 0.979 [95% CI = 0.963–0.995]) showed lower risk of increased BMI, but the advantage does not successfully radiate significant obesity consequences. In addition, the increased density of bus stops can also reduce the risk of obesity in women groups (*p* = 0.035; OR = 0.910 [95% CI = 0.836–0.990]). These findings suggest that women were more sensitive and were more likely to make different behavioral choices and physiological responses due to distinct walking environments. This provides useful evidence for future obesity prevention and urban planning.

## 1. Introduction

The consequences of obesity have been well documented [1,2,3]. Cumulative evidence indicates that obese individuals are at greater risk of some chronic diseases, especially type II diabetes [4,5], cardiovascular diseases [6], obstructive sleep apnea [7], and cancer [8]. Potential factors affecting obesity risk have been identified in literature [9,10,11,12]. In particular, walking environment [13,14] has received considerable attention due to changeability leading to health weight. Different walking environments affect the incidence of obesity by indirectly influencing residents’ physical exercise, as well as walking behaviors, a notion widely recognized by academia [15].

**Figure 1:**
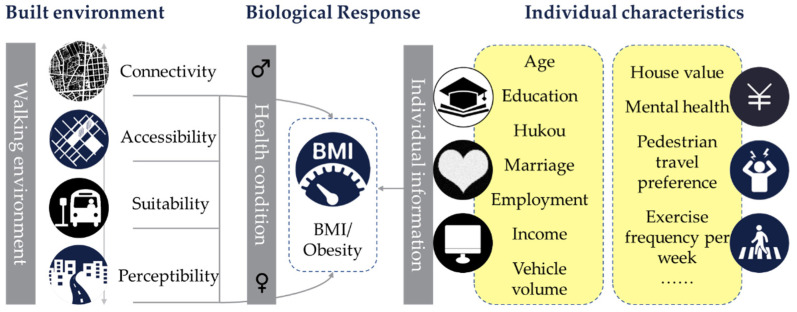
Research framework.

However, limited information is known about whether the walking environment affects the risk of obesity indiscriminately in different social groups. Understanding which populations might be most vulnerable to the obesogenic environment is of great significance for improving urban built environments and targeting obesity prevention interventions. Taking gender as an example, men and women have certain disparities in the scope, frequency, and perceived experience of the walking environment [16,17,18]; thus, the behavioral choices they make and the opportunities and risks they face in the same construction might vary greatly [19,20]. Therefore, men and women are often exposed to obesity-driven risk factors to varying degrees, thereby forming different biological responses (Figure 1). Although the mechanism of the walking environment’s influence on obesity is relatively clear, the interaction among gender differences, walking environment, and incidence of obesity has been rarely investigated. Hence, this article will focus on the effect of gender differences on the obesity risk of residents affected by the walking environment and explore different underlying mechanisms.

A great quantity of literature mentions that women are much less likely to use walking environments and public spaces than men [16], both in terms of time and space. Women’s increased concerns about safety in their daily lives also make their use of open spaces such as streets less frequent than men’s [21,22]. Such unfair access to public space resources grants men greater discourse power in urban planning. Urban planning [23,24] that caters to males also grants men greater activity space and frequency of use in the built environment than women, further consolidating their dominant position. Thus, we can infer that: The impact of walking environment on BMI index and obesity risk in men should be more significant than that in women. Women are less affected by the walking environment due to their lower use of public space; men, on the other hand, spend more time in open spaces and experience more of the walking environment around the neighborhood, which also has a deeper impact on their behavioral choices.

Since men and women are often exposed to obesity-driven risk factors to varying degrees, the present study will focus on the gender differences in the obesity risk of residents affected by the built environment and explore the different mechanisms of its impact on obesity. We constructed a collection of walking environmental indicators by information and communication technology. Then, we prepared a resident health sampling questionnaire to assess the health status and individual characteristics of interviewees. Finally, two sets of models were established using the generalized estimation equation. According to the total sample, male sample, and female sample, the impact mechanism of the walking environment on the risk of obesity of residents was explored. Similarities and differences in the obtained results were also analyzed and discussed. Suggestions are put forward for local urban planning.

## 2. Materials and Methods

### 2.1. Study Sample

**Figure 2:**
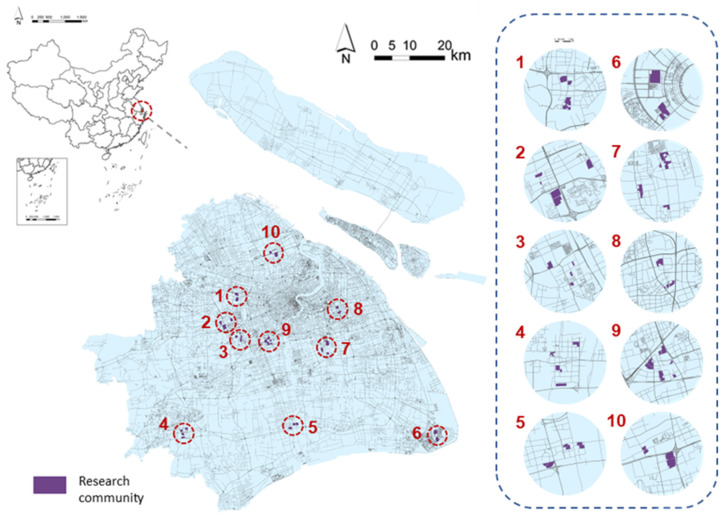
Location of study area.

“Daily Activities and Travel Survey of Shanghai Residents” was conducted in 2017 by using the method of cluster sampling survey from residents living in 54 communities from eight administrative regions of Shanghai (including Jiading District, Fengxian District, Baoshan District, Songjiang District, Pudong New Area, Jinshan District, Minhang District, and Qingpu District) (Figure 2). We have strictly defined the interviewees: (1) the parents’ and children’s (if any) BMI should not be greater than 30 to avoid the genetic impact obesity; (2) the respondents should live in the current residential area for more than 5 years to fully reflect the impact of the built environment; (3) the average respondent should spend less than 6 h a day sitting between 9:00–17:00 daily; and (4) The respondents are neither heavy smokers nor alcoholics. The missing data were omitted from the original participants (*n =* 1479). Finally, 718 men and 637 women (*n =* 1355) were selected to constitute the analysis sample (91.6% of the original sample).

Scholars have argued that Shanghai should be an ideal example to examine typical global issues. Located in the Yangtze River Delta, Shanghai is one of the most developed megacities in China, with an area of 6340 km^2^ and a population of 24.24 million people. Having experienced amazing socio-economic development and large-scale urban expansion in the past few decades, Shanghai has profoundly recognized the significance of people’s health during the development process. To this end, the local government has initialized a new healthy city plan, which emphasizes the optimization of the built environment and equal enjoy rights. In terms of the walking environment, residents should make full use of the park, forest belt, roof, and other green spaces along the river; and take 15 min of walking as the scale of community living space, closely around the clothing, food, housing, and transportation, forming a life circle for community residents. Moreover, the walking environment of Shanghai blocks may vary greatly due to historical legacy (the concession area became a state that was not administered by the local government), thereby providing a typical case for studying the walking environment. In order to eliminate the influence of food environment confounding factors, we measure the food environment of the sampled communities (Supplementary description S1). After the statistics of the food accessibility index of each community, we found that the food accessibility index of different communities was about 2.89, with a small standard deviation of 1.34. Therefore, there is no significant difference in the food environment exposure between samples.

### 2.2. Key Study Variables

#### 2.2.1. Outcome Variables

Resident weight status. Residents were assessed by trained technicians at the collection centres as the participants wore light clothes and no shoes. Anthropometrics’ body weight and height were measured twice at each interview, using a digital scale recording to the nearest 0.1 kg and the Seca 202 device recording to the nearest 0.1 cm, respectively. The two height & weight measurements were averaged if the difference between them is within the accidental error. Otherwise, the measurement nearer to the median weight/height for that age was retained. The measured weight and height were used to calculate BMI (kg/m^2^) for each resident. We also define another outcome variable named “Obesity index” as an ordered Logistic function. In this variable, overweight was defined as BMI ≥ 25 kg/cm^2^, and obesity was supposed to BMI ≥ 30 kg/cm^2^. In our sample, 83.6% of residents had a BMI of less than 25 kg/m^2^, showing a relatively healthy state. 16.4% of residents were considered an alarming health risk, i.e., their BMI was greater than or equal to 25 kg/m^2^. Among this group, 1.3% of residents were already obese, with a BMI of 30 kg/m^2^ or higher.

#### 2.2.2. Exposure Variables: Walking Environment

Our studies followed various frameworks, such as 5C [25], 7C [26], 3D [27], 5D [28], SPCES [29], IMI [30], PEDS [31], and SP [32], to guide the selection and structuring of street walkability indicators. These frameworks have basically the same meaning, but different ways of expressing it. We first collected the definitions and expression of all the components in the framework and then used natural language processing for word frequency analysis. Words with frequency higher than the 30% quantile threshold are retained. Experts from related industries (e.g., Urban Planning Bureau, Traffic Management Bureau, etc.) were invited to participate in the evaluation process. Experts rated each item by judging the measurability, discernibility, verifiability and scale suitability of each item on a scale of 1 (very unsuitable) to 5 (very suitable). Then calculate the average score of each item by assigning equal weights to all criteria and experts, and items were discarded when one or more experts assigned very low values to them or indicated that they were grossly inappropriate. We further invited experts to judge the classification and classified the preserved items as the main component.

After several rounds of feedback, a consensus on the classification was reached, finally forming the walking environment evaluation index system (Table S1). More specifically, the framework consisted of four fundamental components (including connectivity [33,34], accessibility [35,36], suitability [26,37], and perceptibility [38]), as well as seven more detailed indexes that were selected from 12 indexes after the collinearity test, containing road intersection density, land use mix index, number of bus stops within 500 m of the community border, Ratio Vegetation Index (RVI), community green space rate, road green view index, and road sky view index. Among them, RVI is a sensitive indicator parameter for green plants for detecting and estimating plant biomass. All of these indexes were constructed at a community level and were the finest geographic unit at which walking environments have been associated with residents’ outdoor physical activity. In order to describe the walking environment more accurately, five national geographic datasets available in 2017 were used across Shanghai in a GIS environment, including the second national land survey data, remote sensing image data, POI datasets, road network datasets, and street view datasets.

#### 2.2.3. Covariates

Socio-demographic covariates comprised gender, age, education, hukou, marriage, employment, income, house values, housing property, exercise frequency per week, pedestrian travel preference, and vehicle volume. Among them, income (including the sum of wages, pensions, subsistence allowances, other non-wage income, etc.), house value, vehicle volume, along with exercise frequency per week, were assessed from self-reported data. Residents’ marital status was defined as married, unmarried, divorced, and widowed. Self-reported information on highest degree obtained was used to classify participants into six education categories: primary school and below, junior high school, senior school (including polytechnic school and vocational high school), college, university, bachelor’s or higher. Employment status included full-time employment, half-time employment, temporary employment, school students, retired at home, unemployed, and others. Respondents reported their Hukou as falling into one of four categories: Shanghai non-agricultural household hukou, Shanghai agricultural household hukou, nonlocal non-agricultural household hukou, and non-local agricultural household hukou. Housing property was recorded into two categories: head of household and non-head of household. Similarly, pedestrian travel preference was also grouped into five categories due to questionnaire answers, including very dislike, relatively dislike, normal, relatively like, and very like.

### 2.3. Statistical Analyses

In this study, we conducted the LSD Multiple Test on various indexes of walking environments. Furthermore, chi-square analysis was used to test differences in individual characteristics of male and female residents, while the heterogeneity test was used to examine the differences between walking environment of men and women. After the collinearity test, no evidence of multicollinearity (VIF < 10) was found. The statistical significance was set to α < 0.05.

We identified important gender differences in the relationship between obesity (BMI) and walking environment by using generalized estimation equation (GEE) with the clustering of sample selection at the community level. This study evaluated two models in total. These two models demonstrated the impact mechanism of walking environment on weight gain or obesity, respectively, with BMI/Obesity index as the dependent variables and various indicators measuring walking environment as the independent variables. We adjusted for covariates (age, gender, marital status, education, Hukou, employment, income, house value, housing property, exercise frequency per week, pedestrian travel preference, and vehicle volume) that could be potential confounders or predictors of obesity. Among them, exercise frequency per week and the amount of vehicle volume were included in the sensitivity analysis as additional factors. Then, the OR value and its 95% confidence interval were calculated while exploring its significance. All operations are performed in SPSS 25.0 and Stata 12.0.

Each model evaluated three samples: Sample 1 included all participants, while only male residents were included in Sample 2 and only female residents were included in Sample 3. The model formula of the generalized estimation equation is as follows:

(1) $$E\left(Y_{ij}\right)=\mu_{ij}$$

(2) $$g\left(\mu_{ij}\right)=\beta_{0}+\beta_{walk}X_{walkij}$$

where g is the connection function, and $\beta_{0}$ and $\beta_{walk}$ are parameter vectors to be estimated by the model; $Y_{ij}$ is the BMI index or Obesity index measured by the *j*-th individual in the *i*-th community; and $X_{walkij}$ is the walking environment index group corresponding to $Y_{ij}$.

(3) $$X_{ijwalk}=\left(C_{ij},\;A_{ij},\;S_{ij},\;P_{ij}\right)$$

## 3. Results

### 3.1. Descriptive Statistics

The sociodemographic and health status of the samples are listed in Table 1. Males and females show significant differences among BMI index (*p* < 0.001), education level (*p* < 0.001), marital status (*p* < 0.001), employment conditions (*p* < 0.001), and exercise frequency per week (*p* = 0.025). The BMI index of male samples is significantly greater than that of females, which are 22.97 and 21.44, respectively. In terms of employment conditions, the ratio of full-time employment of male residents is significantly higher, and the number of retired and unemployed individuals is relatively small. The proportion of women with higher education was significantly lower, especially up to 5.97% of the females have only primary school education or below. In the aspect of marital status, more women are married and widowed than men. Additionally, male and female residents exercised 3.16 times and 2.91 times a week, indicating that males are significantly higher than females. Although no significant differences in other individual characteristics were found, the average age of the female sample is about 1 year older than that of the male, and the overall income level of the male is slightly higher; compared with male residents, females are often more willing to travel on foot.

**Figure 3:**
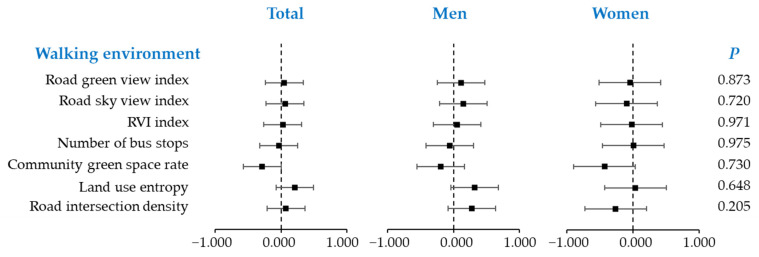
The heterogeneity of the built environment between men and women.

The heterogeneity of the walking environment exposed by the participants of different genders are listed in Figure 3. The results showed that even though men and women experience a certain degree of differences in walking environment, there is no significant heterogeneity between them. This is consistent with our guess; men and women are usually randomly distributed in distinct communities.

**Figure 4:**
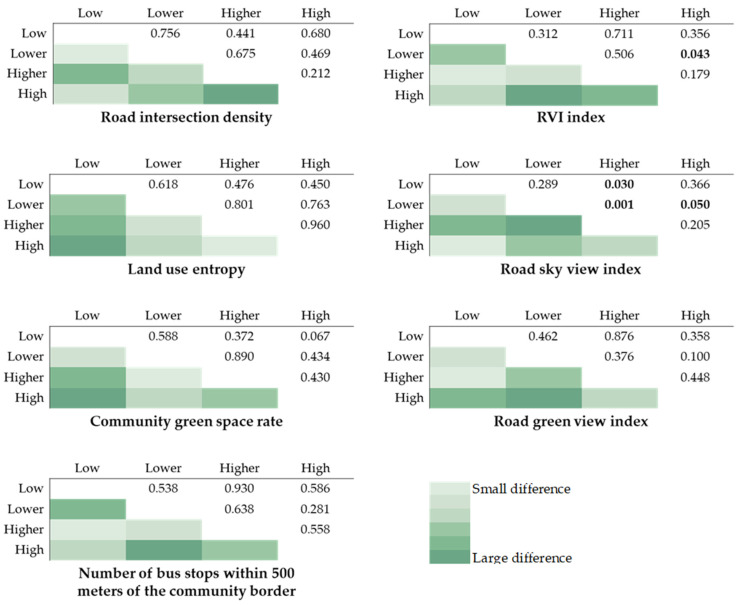
Multiple tests between each level in the built environment.

We further analyzed the discrepancies between each level of walking environment (Figure 4). The quartiles are used to divide the walking environment indexes into four levels: low, lower, higher, and high. Only the RVI index of the lower and higher levels is significant (*p* = 0.043), indicating that a large difference exits in the risk of obesity among residents of these two levels. Besides, more levels in the road sky view index, such as low level and higher level (*p* = 0.030), lower level and higher level (*p* = 0.001), lower level and high level (*p* = 0.050) have significant differences, showing that different grades of streets blue space have a greater impact on the shape of the residents.

### 3.2. Gender Differences in Weight Gain

In Model 1 with the BMI index as the dependent variable, for the total sample of *n* = 1355, the cross-sectional correlations between all indicators of walking environment and BMI index are relatively small and not statistically significant (Table 2). This might be due to sample selections, because the mechanism of the impact of walking environment on residents’ weight gain is diverse, and men and women often show different reactions in different walking environments. Therefore, we divided the total sample into male sample and female sample and analyzed the different results between them.

The sample of male residents (*n* = 718) shows that the association between all walking environment indicators and obesity remains non-significant; while the female sample (*n* = 637) shows that the road intersections density (*p* = 0.009; OR = 0.979 [95% CI = 0.963–0.995]) and road sky view index (*p* = 0.033; OR = 0.002 [95% CI = 0.001–0.619]) are significant, indicating that a higher road network topology and spacious road sky view have a positive effect on the reduction of the BMI index of female residents, which is evidently different from the male sample and the total sample. Surprisingly, a higher RVI index was significantly associated with an increase in BMI among female residents (*p* = 0.050; OR = 1.641 [95% CI = 1.001–2.692]). This might be due to a better vegetation index often representing a more canopied and insecure environment [39,40]. Also, women are more concerned about security [41,42], so they often feel more anxious and fearful in such spaces.

After considering individual characteristics, we can see that gender differences have a significant effect on the risk of weight gain in the total sample (*p* = 0.000; OR = 5.352 [95% CI = 3.866–7.410]), which is the basis to further explore the gender differences in the impact of built environment on obesity. In addition, the influence of age difference on weight gain is significant in all samples. Compared to young people, the elderly are more likely to be overweight. However, retired residents show a significant relationship with weight gain, indicating that they are less at risk of weight gain. Low resident income is also one of the significant risk factors for BMI increase, especially for female samples (*p* = 0.000).

### 3.3. Gender Differences in Obesity

In Model 2 with the “Obesity index” as the dependent variable (Table 3), as far as walking environment, all the indicators still do not show a significant statistical relationship with the risk of obesity for the total and the male samples, and yet in the female sample, number of bus stops within 500 m of the community border turns significant, showing that with a number of bus stops within 500 m of the community border increasing, the likelihood of residents being obese is reduced to 91.0% (*p* = 0.035; OR =0.910 [95% CI = 0.836–0.990]). This result indicates that improving the accessibility of bus stops has a positive significance for reducing the risk of obesity in women.

Taking the individual characteristics into account, in the total and the female samples, residents who are unmarried have a lower likelihood of obesity (9.1% risk of obesity in total sample and 2.2% risk in the female sample) than those who are widowed. Similarly, with the increase of age, both men and women have an increased risk of obesity, which shows that for every one-year increase in age, men are 1.030 times more likely to be overweight or obese, while women are 1.063 times the original. Lower income is also one of the risk factors for women’s obesity, which is represented in the probability of female obesity being 96.8% when the income increases by one unit.

### 3.4. Sensitivity Analysis

**Figure 5:**
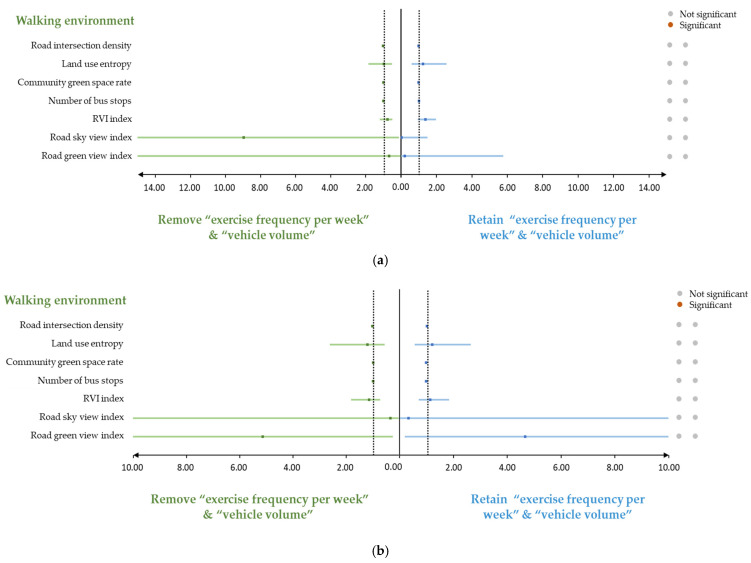

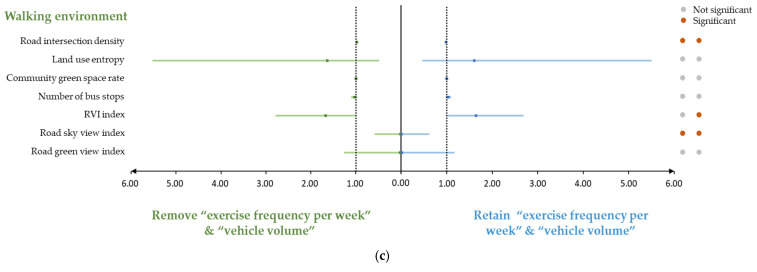
Sensitivity analysis. (**a**) Sensitivity analysis of the total sample; (**b**) Sensitivity analysis of the male sample; (**c**) Sensitivity analysis of the female sample.

After incorporating “exercise frequency per week” and “vehicle volume” into the sensitivity analysis, we have found that in the total sample and the male sample, all walking environment indicators show no significant differences (i.e., from significant to insignificant, or from insignificant to significant). However, in terms of female sample, after excluding the two indicators mentioned before, the RVI index also changes from significant to insignificant (Figure 5).

## 4. Discussion

Our research has achieved different results from previous studies and our conjecture as for the walking environment. Previous studies reported that male residents tend to use open spaces more frequently than women and are exposed to walking environments for longer periods of time; as such, a suitable walking environment could better promote men’s lifestyle of physical exercise [43] and reduce the incidence of obesity. Moreover, the relationship between men and neighborhood walking environment is more consistent with the negative correlation of BMI than women [44,45].

Our research, in contrast, shows that it is women who are more sensitive to the walking environment. First, one possible reason for the opposite result is that fewer Chinese women have driver’s licenses than men. In the data released in 2017, the number of men holding driver’s licenses accounted for 71.21% of the total number of drivers, which was significantly higher than that of women. The results also show that men’s income level is generally higher (Table 1), and they have better economic ability to support a car. Even if men and women in a family hold driver’s licenses, men tend to use cars more frequently than women.

Second, environmental factors related to neighborhood walking ability may also be risk factors for obesity [46]. For example, walkability has been linked to higher levels of air pollution and traffic noise exposure due to traffic congestions and greater concentration of vehicle traffic [47], which may increase the risk of obesity [48,49,50]. Therefore, given that men tend to use more urban space than women, their negative impact on obesity caused by exposure to air pollution, traffic noise, and other risk factors is offset by the positive impact of more walking and physical activity on reducing obesity risk. Thus, the final result is shown as insignificant, which was also confirmed in a recent study in London [51]. Therefore, a strategy to promote health along one path may have an adverse effect on another. The interactions between them should be understood to formulate more effective health promotion strategies and avoid unexpected adverse health consequences.

Third, compared with men, women pay more attention to the comfort and spaciousness of walking spaces. Women often look forward to having a safe and enjoyable experience in the outdoor environment and seek social resources and/or space for rehabilitation [52], especially for women who have formed their families. They regard walking activities during leisure time as restorative “self-time” [53] and have higher sensitivity to the blue and green spaces used during walking. Finally, this finding can also be attributed to the analysis sample. The average age of the female sample in this study is higher than that of the male sample, so the influence of age on the risk of obesity between men and women should also be considered.

In addition, female residents are more sensitive to physical activity and vehicle volume than males. After adding “exercise frequency per week” and “vehicle volume” to the model, the originally insignificant number of RVI index become significant. From a physiological point of view, obesity plays an important role in female reproduction, which is not so important for men. Therefore, women’s basal metabolic rate is lower, which means that they burn less calories at rest and need more physical activity to balance. Moreover, driving behavior is a sedentary behavior that is undertaken outside the home [54], and is a determinant of weight status and poor health [55]. Consequently, controlling the impact of physical activity and vehicle volume is more important in female samples.

This study also has three limitations. First, the walking environment may change with the seasons, and the effect of plants’ autumn colors, fallen leaves, and bare branches may be completely different from that of the lush period in summer. Second, our study is only a cross-sectional sample, and the scale of obesity cases is not large. Studies using larger samples, longer follow-up periods, and more accurate measurements of the community environment are needed to further elucidate the effects of walking environments on the risk of obesity among residents. In particular, isolating interpersonal relationships from socioeconomic effects to improve our understanding of the mechanisms of obesity in men and women should be the focus in future research. Third, smoking is also one of the important risk factors affecting obesity, but our study did not take it into account. However, the level of education has a significant impact on whether an individual smokes or not and incorporating it into the model can slightly alleviate the effects of smoking.

## 5. Conclusions

The present study included gender differences in the mechanism of the built environment’s impact on obesity, revealing the relationships between built environments and residents’ BMI and obesity risk using the questionnaire data of the “Daily Activities and Travel Survey of Shanghai Residents” in 54 communities in Shanghai. It suggests that: (1) women are more susceptible to the obesogenic factors than men in the aspect of the walking environment; (2) improving the topology of roads around the community and ameliorating the comfort of walking spaces have a significant impact on reducing the risk of female weight gain; and (3) improving the accessibility of bus stops will decrease women’s risk of obesity.

The contribution of this study is to increase the empirical analysis that the association between the residential walking environment and change in obesity might be dependent on gender, and to demonstrate that different genders have different sensitivity levels to various indicators of walking environment. This study is the first to put forward the distinct effects of gender differences in the walking environment on residents’ perception experience and behavioral choices. The theoretical framework of the walking environment that affects obesity prevalence has been improved, providing a theoretical reference for policy makers.

The policy implications of this article are that ameliorating the walkability of streets may be important public health interventions to reduce the obesity prevalence of the Shanghai population in the future (especially the female population). A large skeleton of walkable roads blocked the continuity between various functional activities of the city, which was related to the reduction in physical activities [56]. For female residents, completely separating work and residence is difficult. The working space of female residents is not only the employment space, but also the living space. Therefore, female residents need more community environments with a rich road network. In addition, the road landscape should be perfected, and the comfort of the walking environment should be enhanced. In conclusion, improving the density of road intersections, emphasizing the construction of neighborhood and community facade landscape, and stimulating the vitality of urban space are important to increase the livability of the city and ultimately affect the health and well-being of residents.

## Figures and Tables

**Table 1:** Individual characteristics and differences between male and female participants (*n* = 1355).

Characteristic	Total (*n* = 1355)	Male (*n* = 718)	Female (*n* = 637)	*p* ^a^
BMI/kg/m^2^ (mean (SD))	22.25 (2.92)	22.97 (2.81)	21.44 (2.84)	**<0.001 ^b^**
Age/year (mean (SD))	38.75 (11.42)	38.02 (11.51)	39.58 (11.28)	0.081
Education (*n* (%))				
Primary school and below	51 (3.76%)	13 (1.81%)	38 (5.97%)	**<0.001**
Junior high school	258 (19.04%)	116 (16.16%)	142 (22.29%)	
Senior school (including polytechnic school and vocational high school)	325 (23.99%)	189 (26.32%)	136 (21.35%)	
College	273 (20.15%)	148 (20.61%)	125 (19.62%)	
University	382 (28.19%)	214 (29.81%)	168 (26.37%)	
Bachelor’s or higher	66 (4.87%)	38 (5.29%)	28 (4.40%)	
Hukou (*n* (%))				
Shanghai non-agricultural household hukou	650 (47.97%)	353 (49.16%)	297 (46.62%)	0.097
Shanghai agricultural household hukou	62 (4.58%)	24 (3.34%)	38 (5.97%)	
Non local non-agricultural household hukou	375 (27.68%)	193 (26.88%)	182 (28.57%)	
Non local agricultural household hukou	268 (19.78%)	148 (20.61%)	120 (18.84%)	
Marriage (*n* (%))				
Married	1077 (79.48%)	537 (74.79%)	540 (84.77%)	**<0.001**
Unmarried	262 (19.34%)	172 (23.96%)	90 (14.13%)	
Divorced	11 (0.81%)	8 (1.11%)	3 (0.47%)	
Widowed	5 (0.37%)	1 (0.14%)	4 (0.63%)	
Employment (*n* (%))				
Full-time employment	980 (72.32%)	573 (79.81%)	407 (63.89%)	**<0.001**
Half-time employment	27 (1.99%)	13 (1.81%)	14 (2.20%)	
Temporary employment	16 (1.18%)	9 (1.25%)	7 (1.10%)	
School students	48 (3.54%)	32 (4.46%)	16 (2.51%)	
Retired at home	141 (10.41%)	45 (6.27%)	96 (15.07%)	
Unemployed	138 (10.18%)	42 (5.85%)	96 (15.07%)	
Other	5 (0.37%)	4 (0.56%)	1 (0.16%)	
Housing property (*n* (%))				
Head of household	869 (64.13%)	436 (60.72%)	438 (68.76%)	0.650
Non-head of household	486 (35.87%)	282 (39.28%)	199 (31.24%)	
Pedestrian travel preference (*n* (%))				
Very dislike	25 (1.85%)	12 (1.67%)	13 (2.04%)	0.118
Relatively dislike	135 (9.96%)	75 (10.45%)	60 (9.42%)	
Normal	529 (39.04%)	299 (41.64%)	230 (36.11%)	
Relatively like	516 (38.08%)	263 (36.63%)	253 (39.72%)	
Very like	150 (11.07%)	69 (9.61%)	81 (12.72%)	
House value/RMB (mean (SD))	3,105,758.18 (1,825,849.11)	3,062,767.56 (1,908,874.76)	3,154,215.43 (1,727,703.53)	0.589
Vehicle volume (mean (SD))				
Number of cars	0.65 (0.61)	0.65 (0.61)	0.64 (0.61)	0.278
Number of electric vehicles /mopeds /motorcycles	0.61 (0.68)	0.64 (0.69)	0.59 (0.66)	0.393
Number of bicycles	0.44 (0.65)	0.43 (0.68)	0.45 (0.62)	0.813
Exercise frequency per week/Times (mean (SD))	3.04 (2.99)	3.16 (3.04)	2.91 (2.93)	**0.025**
Income/RMB (mean (SD))	15,727.07 (21,847.11)	15,892.76 (20,947.84)	15,540.31 (22,833.46)	0.232

^a^ significant result expressed as chi-square test. ^b^ bold text indicates statistical significance (*p* < 0.05).

**Table 2:** Generalized linear estimation equations testing for the increased BMI among residents based on walking environment.

Model 1	Total Sample		Male Sample		Female Sample	
	*p*-Value	OR (95% CI)	*p*-Value	OR (95% CI)	*p*-Value	OR (95% CI)
Walking environment						
Road intersection density	0.227	0.994 (0.985, 1.004)	0.104	1.008 (0.998, 1.019)	**0.009**	**0.979 (0.963, 0.995)**
Land use entropy	0.559	1.241 (0.602, 2.558)	0.633	1.210 (0.553, 2.647)	0.455	1.601 (0.466, 5.504)
Community green space rate	0.375	0.988 (0.962, 1.015)	0.277	0.983 (0.952, 1.014)	0.838	0.996 (0.955, 1.038)
Number of bus stops within 500 m of the community border	0.490	1.014 (0.975,1.055)	0.601	0.986 (0.936, 1.039)	0.305	1.034 (0.970, 1.101)
RVI index	0.099	1.368 (0.943, 1.984)	0.586	1.140 (0.711, 1.830)	**0.050**	**1.641 (1.001, 2.692)**
Road sky view index	0.079	0.031 (0.001, 1.487)	0.638	0.328 (0.003, 34.241)	**0.033**	**0.002 (0.001, 0.619)**
Road green view index	0.358	0.213 (0.008, 5.761)	0.344	4.660 (0.192, 112.86)	0.059	0.012 (0.001, 1.176)
Individual characteristics						
Gender (Ref: Female)						
Male	**0.000**	**5.352 (3.866, 7.410)**	**/**	**/**	/	/
Education	No significant effect shown, see Table S2 for details					
Hukou	No significant effect shown, see Table S2 for details					
Marriage	No significant effect shown, see Table S2 for details					
Employment (Ref: Other)						
Full-time employment	0.154	0.294 (0.055, 1.580)	0.094	0.193 (0.028, 1.322)	0.122	0.441 (0.156, 1.244)
Half-time employment	0.709	0.675 (0.085, 5.329)	0.354	0.221 (0.009, 5.379)	0.651	1.448 (0.291, 7.197)
Temporary employment	0.209	0.306 (0.048, 1.941)	0.318	0.308 (0.030, 3.117)	0.112	0.262 (0.050, 1.370)
School students	0.791	0.773 (0.116, 5.170)	0.812	0.760 (0.080, 7.235)	0.321	0.527 (0.148, 1.871)
Retired at home	**0.027**	**0.165 (0.034, 0.812)**	**0.006**	**0.068 (0.010, 0.471)**	**0.038**	**0.276 (0.082, 0.928)**
Unemployed	0.231	0.339 (0.058, 1.988)	0.136	0.207 (0.026, 1.641)	0.259	0.503 (0.152, 1.660)
Housing property	No significant effect shown, see Table S2 for details					
Pedestrian travel preference	No significant effect shown, see Table S2 for details					
Age	**0.000**	**1.061 (1.040, 1.083)**	**0.000**	**1.060 (1.031, 1.090)**	**0.001**	**1.065 (1.038, 1.093)**
House value	0.668	1.000 (1.000, 1.000)	0.704	1.000 (1.000, 1.000)	0.148	1.000 (1.000, 1.000)
Number of cars	0.616	0.936 (0.722, 1.212)	0.871	0.973 (0.699, 1.355)	0.877	0.970 (0.658, 1.429)
Number of electric vehicles /mopeds /motorcycles	0.280	0.881 (0.699, 1.109)	0.340	0.856 (0.623, 1.177)	0.827	0.956 (0.639, 1.430)
Number of bicycles	0.224	1.143 (0.922, 1.417)	0.213	1.201 (0.901, 1.601)	0.726	1.057 (0.776, 1.439)
Exercise frequency per week	0.381	1.024 (0.972, 1.078)	0.215	1.043 (0.976, 1.116)	0.856	0.991 (0.904, 1.087)
Income	**0.044**	**0.958 (0.800, 0.999)**	0.842	0.977 (0.960, 1.023)	**0.000**	**0.920 (0.902, 0.980)**

Dependent variable: BMI index; Bold text indicates statistical significance (*p* < 0.05).

**Table 3:** Generalized linear estimation equations testing for the risk of obesity among residents based on walking environment.

Model 2	Total Sample		Male Sample		Female Sample	
	*p*-Value	OR (95% CI)	*p*-Value	OR (95% CI)	*p*-Value	OR (95% CI)
Walking environment						
Road intersection density	0.998	0.999 (0.991, 1.009)	0.058	1.011 (1.000, 1.022)	0.066	0.974 (0.946, 1.002)
Land use entropy	0.940	1.025 (0.534, 1.971)	0.417	1.332 (0.666, 2.663)	0.582	0.714 (0.215, 2.372)
Community green space rate	0.548	1.009 (0.979, 1.040)	0.527	1.012 (0.974, 1.052)	0.738	1.007 (0.966, 1.051)
Number of bus stops within 500 m of the community border	0.713	1.009 (0.964, 1.055)	0.237	0.966 (0.913, 1.023)	**0.035**	**0.910 (0.836, 0.990)**
RVI index	0.264	1.307 (0.817, 2.090)	0.555	1.169 (0.697, 1.959)	0.568	1.468 (0.393, 5.488)
Road sky view index	0.387	0.147 (0.002, 11.339)	0.455	0.150 (0.001, 21.657)	0.418	0.021 (0.001, 234.921)
Road green view index	0.846	1.457 (0.033, 64.454)	0.043	54.011 (1.132, 2576.444)	0.123	0.007 (0.002, 3.924)
Individual characteristics						
Gender (Ref: Female)						
Male	**0.000**	**2.799 (1.849, 4.237)**	**/**	**/**	/	/
Education	No significant effect shown, see Table S3 for details					
Hukou	No significant effect shown, see Table S3 for details					
Marriage (Ref: Widowed)						
Married	0.163	0.293 (0.052, 1.646)	**/**	**/**	0.132	0.123 (0.008, 1.877)
Unmarried	**0.008**	**0.091 (0.016, 0.531)**	**/**	**/**	**0.016**	**0.022 (0.001, 0.485)**
Divorced	0.133	0.140 (0.011, 1.814)	**/**	**/**	0.143	0.189 (0.028, 1.856)
Employment	No significant effect shown, see Table S3 for details					
Housing property	No significant effect shown, see Table S3 for details					
Pedestrian travel preference (Ref: Very like)						
Very dislike	0.445	0.624 (0.186, 2.092)	0.977	1.018 (0.306, 3.383)	0.489	0.689 (0.347, 1.784)
Relatively dislike	0.190	0.619 (0.302, 1.269)	0.339	0.657 (0.278, 1.554)	0.735	0.810 (0.240, 2.737)
Normal	**0.035**	**0.591 (0.362, 0.964)**	0.146	0.604 (0.307, 1.192)	0.511	0.771 (0.355, 1.676)
Relatively like	0.175	0.708 (0.430, 1.166)	0.294	0.698 (0.357, 1.365)	0.986	0.992 (0.405, 2.430)
Age	**0.019**	**1.024 (1.004, 1.044)**	**0.009**	**1.030 (1.007, 1.054)**	**0.002**	**1.063 (1.023, 1.105)**
House value	0.863	1.000 (1.000, 1.000)	0.534	1.000 (1.000, 1.000)	0.209	1.000 (1.000, 1.000)
Number of cars	0.341	0.864 (0.640, 1.167)	0.546	0.896 (0.629, 1.278)	0.718	0.891 (0.478, 1.664)
Number of electric vehicles /mopeds /motorcycles	0.448	0.915 (0.728, 1.151)	0.577	0.929 (0.717, 1.204)	0.670	0.906 (0.576, 1.426)
Number of bicycles	0.245	1.158 (0.904, 1.483)	0.174	1.198 (0.923, 1.555)	0.261	1.304 (0.821, 2.070)
Exercise frequency per week	0.168	1.036 (0.985, 1.090)	0.338	1.198 (0.923, 1.555)	0.399	1.047 (0.941, 1.166)
Income	0.489	0.980 (0.934, 1.002)	0.952	0.965 (0.946, 1.069)	**0.008**	**0.968 (0.947, 0.974)**

Dependent variable: Obesity index; Bold text indicates statistical significance (*p* < 0.05).

## Data Availability

The “Daily Activities and Travel Survey of Shanghai Residents” questionnaire data are not publicly available due to protection of privacy of the participants by all means. The Shanghai Road Network dataset was obtained from 2017 Gaode Map (https://www.amap.com) (accessed on 7 July 2021). POI data was obtained from 2017 Baidu map (https://map.baidu.com) (accessed on 7 July 2021). For remote sensing image data of Shanghai, the LANDSAT 7 ETM+ (15 m) image of Shanghai in August 2017 was collected for free from the U.S. Geological Survey (https://www.usgs.gov/) (accessed on 7 July 2021) in the United States. The Shanghai Street View Dataset was obtained from 2017 Baidu map (https://map.baidu.com) (accessed on 7 July 2021).

## Supplementary Materials

The following supporting information can be downloaded at: https://www.mdpi.com/article/10.3390/ijerph19042056/s1. Table S1: Selection of indicators for measuring the walking environment. Table S2: Generalized linear estimation equations for testing the increased BMI among residents (Connected to Table 2). Table S3: Generalized linear estimation equations for testing the risk of obesity among residents (Connected to Table 3).
