# Supplementary material for: Walking Environment and Obesity: A Gender-Specific Association Study in Shanghai
Source: Int J Environ Res Public Health. 2022 Feb 12;19(4):2056. doi: 10.3390/ijerph19042056 (PMC8871922; doi:10.3390/ijerph19042056)
Supplement: Supplementary file 1 [file ijerph-19-02056-s001.zip › ijerph-1593716-supplementary.pdf]

Supplementary materials

Supplementary description S1: We propose a two-step floating catchment area (2SFCA) method to measure food environment, in this connection food accessibility and health are focused. Accessibility refers to the convenience of getting from a community to a provisions shop, generally expressed in terms of the road network distance (D), while health is mainly concerned with the type of food. Compared with Europe and the United States, Chinese cuisine has a long history, especially with abundant variety, exquisite material, excellent craftsmanship and has profound cultural connotation. Due to differences in geography, climate, products, culture, and beliefs, the flavors of dishes differ greatly, and they have developed into many unique schools. The health index (H) can be obtained by judging and assigning the health of major cuisines in food stores through the evaluation website.

Table S1. Selection of indicators for measuring the walking environment.

Component [ab]	Connotation/Explanation	Data Sources	Index		Equation
Connectivity (C)	Connectivity refers to the degree to which routes are interconnected within the network as well as the extent of various and directional links from origins to destinations. Connectivity is the basic walking function that controls walking activities because it creates direct and indirect routes and options to destinations [33,34].	The Shanghai road Network dataset including road name, road level, road length, road height relative to the ground, and road space location was obtained from 2017 Gaode Map (https://www. amap.com) (accessed on July 7, 2021). Considering that the original data contain all kinds of routes, we deleted the highways, ramps, national roads, provincial roads, expressways, and elevated highroads that do not allow walking, and only the walkable routes are retained.	Road intersection density (RID)		Road intersection density is represented by the weighted density of road intersections within the 1500 m buffer zone around the community. The intersections with high weights show good connectivity, which is related to the number of connected roads and their grades. The specific formula is: $RID_i = \sum_{n=1}^{N_i} \frac{R_n}{\sum_r^{R_n} \frac{Type_{nr}}{R_n}}$ (A1) where *i* is the community code; N_i is the total number of intersections within 1500 m around the community *i*; R_n represents the number of roads passing through intersection *n*; and $Type_{nr}$ refers to the grade of road *r* passing through intersection *n*.
Accessibility (A)	Accessibility represents the degree to which walking has superior efficiency over other modes of transportation. It is closely associated with mixed land use for increasing the access to destinations on feet [35,36].	The second national land survey data of Shanghai included location, scope, area, and use type of land. In this study, ArcGIS was used to calculate the land areas of different use types of 12 first class types (cultivated land, garden land, woodland, grassland, commercial land, residential land, industrial and mining storage land, public management and public	Land use mix [c]	Land use mix index (M)	$M_i = \sum_{n=1}^{N_i} P_n \ln\left(\frac{1}{P_n}\right)$ (A2) *i* is the community code; N_i is the total number of land use types within 600 m around the community *i*; and P_n is the proportion of land use *n*.
				Land use entropy (D)	$D_i = 1 - \sum_{n=1}^{N_i} (P_n)^2$ (A3) i is the community code; N_i is the total number of land use types within 600 m around the community i; P_n is the proportion of land use n

		service land, transportation land, water area and water conservancy facility land, special land, and other land) and 74 s class types in Shanghai. The proportion of such land was further obtained. Finally, land use mix index and land use entropy were calculated by combining with the formula (2 & 3).			
		POI data was obtained from 2017 Baidu map (https://map.baidu.com) (accessed on July 7, 2021). The name of bus stops and latitude and longitude of its spatial location were included.	Number of bus stops within 500 m of the community border		/
Suitability (S)	Suitability denotes the extent to which the characteristics of the street and amenities can ease the walking experience, which is typically correlated with greenspaces, shade, air quality, and cultural landscapes [26,37].	For remote sensing image data of Shanghai, the LANDSAT 7 ETM+ (15 m) image of Shanghai in August 2017 was collected for free from the U.S. Geological Survey (https://www.usgs.gov/) (accessed on July 7, 2021) in the United States. This image was used to calculate various vegetation indices in the study area in ENVI.	Green space quality [d]	NDVI index	$NDVI = \frac{NIR-R}{NIR+R}$ (A4) NIR is the reflection value in the near-infrared band, and R is the reflection value in the red band. NDVI reflects the background influence of plant canopy
				SAVI index	$SAVI = \frac{(NIR-R)(1+L)}{(NIR+R+L)}$ (A5) NIR is the reflection value in the near-infrared band, R is the reflection value in the red band; L is a parameter that changes with the vegetation density, whose value range is 0–1. When the vegetation coverage is very high, it is 0; when it is very low, it is 1. In general, 0.5 is selected to reduce the background difference of soil and remove the impact of soil noise. Thus SAVI explains the changes in the optical characteristics of the background and correct the sensitivity to the soil background.
				EVI index	$EVI = 2.5\left(\frac{NIR-R}{NIR+6R-7.5B+1}\right)$ (A6) where NIR is the reflection value in the near-infrared band, R is the reflection value in the red band, and B is the reflection value in the blue band. EVI corrects the effects

				of soil background and aerosol scattering.
			RVI index	$RVI = \frac{NIR}{R}$ (A7)<br>Where *NIR* is the reflection value in the near-infrared band, and *R* is the reflection value in the red band. RVI is for detecting and estimating plant biomass.
			ARVI index	$ARVI = \frac{NIR-(2R-B)}{NIR+(2R-B)}$ (A8)<br>where *NIR* is the reflection value in the near-infrared band, *R* is the reflection value in the red band, and *B* is the reflection value in the blue band. ARVI addresses the effects of atmospheric scattering.
			Community green space rate (CGR)	$CGR_i = \frac{GS_i}{RL_i}$ (A9)<br>where *i* is the community code; GS_i represents the total area of various green spaces within the scope of the community *i*; and RL_i indicates the total area of the community *i*, reflecting the level of green space in the community plane pattern.
Perceptibility (P)	Perceptibility indicates the degree of comfort and safety for pedestrians [38], presenting a subjective image of the street.	The Shanghai Street View Dataset was obtained from 2017 Baidu map (https://map.baidu.com) (accessed on July 7, 2021). A total of 250842 street view photos containing spatial location information was obtained. After excluding the street view of roads that is not allowed to walk, the street view image recognition technology is adopted to identify different elements in the image, such as people, green trees, buildings, and sky based on the neural network framework, mainly calculating the pixel ratio of green trees/sky. During the training and evaluation phase, the Cityscapes dataset is used (https://www. cityscapes-dataset.com/dataset-	Road green view index (GVI)	$GVI_i = \frac{\sum_{n=1}^{N} GVI_n \cdot L_n}{L_i}$ (A10)<br>where *i* is the community code; *N* represents the number of roads in the 600 m buffer zone around the community *i*; GVI_n is the average green view index of road *n*; L_n is the length of road *n* in the buffer zone; and L_i is the total length of the road in the buffer zone.
			Road sky view index (SVI)	$SVI_i = \frac{\sum_{n=1}^{N} SVI_n \cdot L_n}{L_i}$ (A11)<br>where *N* represents the number of roads in the 600 m buffer zone around the community *i*; SVI_n is the average sky view index of road *n*; L_n is the length of road *n* in the buffer zone; and L_i is the total length of the road in the buffer zone.

overview/) (accessed on July 7, 2021), which includes more than 25,000 images of 50 cities in Europe and their pixel-level semantic annotations as well as 5000 finely labeled and 20,000 nonfinely labeled images. This dataset is composed of street view data for multiple seasons and weather conditions with detailed descriptions in the metadata.

[a] The four components are also closely interlinked with one another. For example, accessibility can be increased through connectivity and suitability, and suitability can also enhance perceptibility. [b] The proposed framework is highly compatible with previous frameworks because the original indicators under these frameworks provide the basis for our framework. For example, the "connectivity" component conforms to the "connection" dimension of the 5C and 7C solutions, and the "accessibility" component accords with the "destination" dimension in SPCES, the "convenient" dimension in the 7C layout, and the "accessibility" dimension in IMI and 5D scheme. [c] After the collinearity test, only land use mix index is retained as an indicator to measure the mixed degree of land use (VIF < 3). [d] After the collinearity test, only the RVI index is selected to measure the quality of green space around the community (VIF < 3).

Table S2. Generalized linear estimation equations for testing the increased BMI among residents (Connected to Table 2).

**Model 1**	**Total Sample**		**Male Sample**		**Female Sample**	
	***p*-Value**	**OR (95% CI)**	***p*-Value**	**OR (95% CI)**	***p*-Value**	**OR (95% CI)**
Education (Ref: Bachelor's or higher)						
Primary school and below	0.649	1.297 (0.423, 3.982)	0.958	1.061 (0.113, 9.928)	0.710	1.329 (0.297, 5.956)
Junior high school	0.122	1.711 (0.867, 3.376)	0.602	1.295 (0.490, 3.422)	0.138	2.344 (0.761, 7.220)
Senior school (including polytechnic school and vocational high school)	0.697	1.124 (0.625, 2.019)	0.945	0.971 (0.429, 2.199)	0.409	1.566 (0.540, 4.540)
College	0.588	1.188 (0.637, 2.215)	0.630	1.237 (0.521, 2.937)	0.781	1.164 (0.399, 3.395)
University	0.617	1.176 (0.623, 2.221)	0.423	1.422 (0.601, 3.366)	0.894	0.931 (0.324, 2.675)
Hukou (Ref: Non local agricultural household hukou)						
Shanghai non-agricultural household hukou	0.443	0.785 (0.423, 1.457)	0.300	0.680 (0.327, 1.411)	0.995	0.997 (0.409, 2.433)
Shanghai agricultural household hukou	0.271	0.592 (0.232, 1.507)	0.368	0.601 (0.199, 1.821)	0.554	0.654 (0.160, 2.668)
Non local non-agricultural household hukou	0.093	0.652 (0.396, 1.074)	0.439	0.753 (0.367, 1.544)	0.136	0.607 (0.315, 1.170)
Marriage (Ref: Widowed)						
Married	0.475	0.261 (0.007, 10.396)	0.659	1.395 (0.317, 6.138)	0.452	0.151 (0.001, 20.819)
Unmarried	0.239	0.110 (0.003, 4.328)	0.526	0.627 (0.148, 2.650)	0.277	0.062 (0.001, 9.410)
Divorced	0.441	0.183 (0.002, 13.786)	0.712	1.597 (0.133, 19.240)	0.261	0.044 (0.001, 10.277)
Housing property (Ref: Non-head of household)						
Head of household	0.141	0.740 (0.496, 1.105)	0.497	0.826 (0.476, 1.433)	0.050	0.586 (0.343, 1.001)
Pedestrian travel preference (Ref: Very like)						
Very dislike	0.692	1.216 (0.462, 3.204)	0.446	1.901 (0.364, 9.926)	0.749	0.841 (0.290, 2.434)
Relatively dislike	0.341	0.716 (0.360, 1.423)	0.326	0.614 (0.232, 1.625)	0.811	1.139 (0.392, 3.306)

Normal	0.381	0.745 (0.386, 1.439)	0.978	1.015 (0.362, 2.845)	0.139	0.578 (0.280, 1.196)
Relatively like	0.388	0.762 (0.412, 1.410)	0.947	0.965 (0.337, 2.766)	0.443	0.739 (0.342, 1.599)

Table S3. Generalized linear estimation equations for testing the risk of obesity among residents (Connected to Table 3).

Model 2	Total Sample		Male Sample		Female Sample	
	p-Value	OR (95% CI)	*p*-Value	OR (95% CI)	*p*-Value	OR (95% CI)
Education (Ref: Bachelor's or higher)						
Primary school and below	0.182	2.188 (0.693, 6.914)	0.316	2.225 (0.467, 10.607)	0.633	0.688 (0.148, 3.199)
Junior high school	0.255	1.679 (0.689, 4.092)	0.292	1.777 (0.610, 5.178)	0.756	0.797 (0.191, 3.333)
Senior school (including polytechnic school and vocational high school)	0.910	1.050 (0.451, 2.445)	0.630	1.290 (0.458, 3.635)	0.236	0.411 (0.095, 1.787)
College	0.751	1.151 (0.484, 2.737)	0.490	1.446 (0.508, 4.113)	0.249	0.439 (0.108, 1.778)
University	0.816	1.102 (0.487, 2.492)	0.409	1.479 (0.585, 3.741)	0.120	0.286 (0.059, 1.385)
Hukou (Ref: Non local agricultural household hukou)						
Shanghai non-agricultural household hukou	0.338	0.793 (0.494, 1.274)	0.152	0.591 (0.288, 1.214)	0.473	1.405 (0.555, 3.557)
Shanghai agricultural household hukou	0.153	0.527 (0.219, 1.269)	0.426	0.595 (0.166, 2.135)	0.354	0.434 (0.074, 2.537)
Non local non-agricultural household hukou	0.162	0.701 (0.426, 1.153)	0.373	0.727 (0.361, 1.465)	0.243	0.627 (0.287, 1.371)
Housing property (Ref: Non-head of household)						
Head of household	0.457	0.860 (0.577, 1.281)	0.596	0.864 (0.503, 1.484)	0.881	0.939 (0.413, 2.134)
Employment (Ref: Other)						
Full-time employment	0.496	0.558 (0.104, 2.995)	0.379	0.482 (0.095, 2.451)	/	/
Half-time employment	0.933	1.084 (0.166, 7.080)	0.822	0.773 (0.082, 7.256)	/	/
Temporary employment	0.626	0.586 (0.068, 5.031)	0.577	0.504 (0.045, 5.619)	/	/
School students	0.453	2.033 (0.318, 12.997)	0.503	1.906 (0.289, 12.586)	/	/
Retired at home	0.221	0.352 (0.066, 1.871)	0.116	0.261 (0.049, 1.394)	/	/
Unemployed	0.769	0.766 (0.129, 4.542)	0.639	0.660 (0.116, 3.752)	/	/
